# Hedgehog components are overexpressed in a series of liver cancer cases

**DOI:** 10.1038/s41598-024-70220-0

**Published:** 2024-08-22

**Authors:** Caroline Brandi Schlaepfer Sales, Rosane Borges Dias, Ludmila de Faro Valverde, Larissa M. Bomfim, Lais Almeida Silva, Nanashara C. de Carvalho, Jorge Luiz Andrade Bastos, Tatiana Martins Tilli, Gisele Vieira Rocha, Milena Botelho Pereira Soares, Luiz Antonio Rodrigues de Freitas, Clarissa A. Gurgel Rocha, Daniel P. Bezerra

**Affiliations:** 1 Department of Biomorphology, Institute of Health Sciences, Federal University of Bahia (UFBA), Salvador, Bahia 40110-902 Brazil; 2 Gonçalo Moniz Institute, Oswaldo Cruz Foundation (IGM-FIOCRUZ/BA), Salvador, Bahia 40296-710 Brazil; 3 Department of Propedeutics, School of Dentistry of the Federal University of Bahia (UFBA), Salvador, Bahia 40110-909 Brazil; 4 Department of Biological Sciences, State University of Feira de Santana (UEFS), Feira de Santana, Bahia 44036-900 Brazil; 5 Department of Dentistry, Federal University of Sergipe (UFS), Lagarto, Sergipe 49400-000 Brazil; 6 Medical School of Bahia, Federal University of Bahia (UFBA), Salvador, Bahia 40110-100 Brazil; 7 Translational Oncology Platform, Center for Technological Development in Health, Oswaldo Cruz Foundation (FIOCRUZ), Rio de Janeiro, Rio de Janeiro 21040-900 Brazil; 8 Laboratory of Cardiovascular Research, Oswaldo Cruz Foundation (FIOCRUZ), Rio de Janeiro, Rio de Janeiro 21040-900 Brazil; 9 D’Or Institute for Research and Education (IDOR), São Rafael Hospital Center for Biotechnology and Cell Therapy, Salvador, Bahia 41650-010 Brazil; 10 SENAI Institute for Innovation in Advanced Health Systems, SENAI CIMATEC, Salvador, Bahia 41650-010 Brazil

**Keywords:** Hepatocarcinoma, Hedgehog proteins, Chemotherapy, Molecular biology, Cancer, Computational biology and bioinformatics, Molecular biology, Gastroenterology, Medical research, Molecular medicine, Oncology, Pathogenesis

## Abstract

Liver cancers, including hepatocellular carcinoma (HCC), are the sixth most common cancer and the third leading cause of cancer-related death worldwide, representing a global public health problem. This study evaluated nine patients with HCC. Six of the cases involved hepatic explants, and three involved hepatic segmentectomy for tumor resection. Eight out of nine tumors were HCC, with one being a combined hepatocellular-cholangiocarcinoma tumor. Conventional markers of hepatocellular differentiation (Hep Par-1, arginase, pCEA, and glutamine synthetase) were positive in all patients, while markers of hepatic precursor cells (CK19, CK7, EpCAM, and CD56) were negative in most patients, and when positive, they were detected in small, isolated foci. Based on in silico analysis of HCC tumors from The Cancer Genome Atlas database, we found that Hedgehog (HH) pathway components (*GLI1*, *GLI2*, *GLI3* and *GAS1*) have high connectivity values (module membership > 0.7) and are strongly correlated with each other and with other genes in biologically relevant modules for HCC. We further validated this finding by analyzing the gene expression of HH components (*PTCH1*, *GLI1*, *GLI2* and *GLI3*) in our samples through qPCR, as well as by immunohistochemical analysis. Additionally, we conducted a chemosensitivity analysis using primary HCC cultures treated with a panel of 18 drugs that affect the HH pathway and/or HCC. Most HCC samples were sensitive to sunitinib. Our results offer a comprehensive view of the molecular landscape of HCC, highlighting the significance of the HH pathway and providing insight into focused treatments for HCC.

## Introduction

Malignant liver tumors, especially hepatocellular carcinoma (HCC), are the third leading cause of cancer-related mortality worldwide and the sixth most frequent type of cancer, representing a global public health problem^1^. There are limited treatment options for HCC due to delays in diagnosis and tumor heterogeneity^2,3^, and consequently, patients have a poor prognosis^4^. Risk factors for HCC development include chronic viral hepatitis, alcohol abuse, environmental carcinogens, and fatty liver disease^1,4^. Most HCC cases occur in cirrhotic livers because of progressive liver damage and fibrosis^5–8^.

Despite liver transplantation (LT) being a well-established treatment for HCC, recurrence is frequent^4,9^. There are several classifications for determining which patients are eligible for LT. According to the Barcelona Clinic Liver Cancer criteria, patients with a single nodule or three nodules less than 3 cm in diameter are classified as having early-stage HCC and are eligible for LT^10^. In many cases, the evaluation of tissues obtained from liver explants can support the determination of the degree of differentiation and presence of microvascular invasion, reflecting a more accurate prognosis and indication of adjuvant strategies^11,12^.

In silico studies in HCC have provided valuable insights into our understanding of the molecular landscape of HCC and have significantly advanced our understanding of disease progression, potential therapeutic targets and other biomarkers^13,14^. The genetic basis of HCC has been correlated with different mutations, including in the TERT promoter, p53, β-catenin, and Axin^15^. The Hedgehog (HH) signaling pathway has also been implicated in the development of HCC, sustained cancer cell growth and progression^16^ and, ultimately, the maintenance of cancer stem cells, resulting in sorafenib resistance^17^.

Furthermore, the complexity of the molecular profile and limited treatment options for HCC have prompted chemosensitivity studies using drugs known for their distinct mechanisms of action, ranging from DNA damage to mitotic inhibition. It can help identify the most effective drugs and overcome drug resistance and the molecular mechanisms underlying drug resistance in HCC^18^.

This study comprehensively analyzed traditional HCC markers in nine patients with this tumor type and evaluated HCC transcriptomics using The Cancer Genome Atlas (TCGA) database with further molecular validation. In addition, we present results from a chemosensitivity assay using primary HCC cells.

## Results

### Clinicopathological features of HCC patients

This study evaluated nine cases of HCC, including tumor (T), lateral margin (TM) at the interface with the nonneoplastic liver, and liver distant from the neoplasm (NNL) samples. Six of the cases involved hepatic explants, and three involved hepatic segmentectomy for tumor resection. Seven patients were male, while two patients were female (Table 1). The age of the patients ranged from 50 to 69 years, with a mean and standard deviation of 63.9 ± 5.7 years. All patients, except for one who had stage F3 fibrosis, had cirrhosis. The most common cause of cirrhosis was hepatitis C virus infection (5 patients), followed by nonalcoholic steatohepatitis (1 patient), alcoholic liver disease (1 patient), Budd Chiari syndrome (1 patient), and cryptogenic cirrhosis (1 patient). There was no evidence of metastasis in any of the patients.

**Table 1:** Clinicopathological data.

Case	Age	Gender	Basic disease	Procedure	Number of tumors	Size of tumors (cm)
HCC5	68	Male	Virus C infection	Liver transplantation	3	2
HCC6	69	Male	Virus C infection	Tumor ressection	1	3.6
HCC7	62	Male	Cryptogenic	Liver transplantation	3	3
HCC8	69	Male	Virus C infection	Tumor ressection	1	2
HCC13	50	Female	Budd-Chiari	Liver transplantation	1	2.2
HCC16	65	Female	Nonalcoholic steatohepatitis	Tumor ressection	1	5.2
HCC17	61	Male	Virus C infection	Liver transplantation	1	1.6
HCC18	63	Male	Alcoolic liver disease	Liver transplantation	3	1.7
HCC22	68	Male	Virus C infection	Liver transplantation	1	4.4

**Figure 1:**
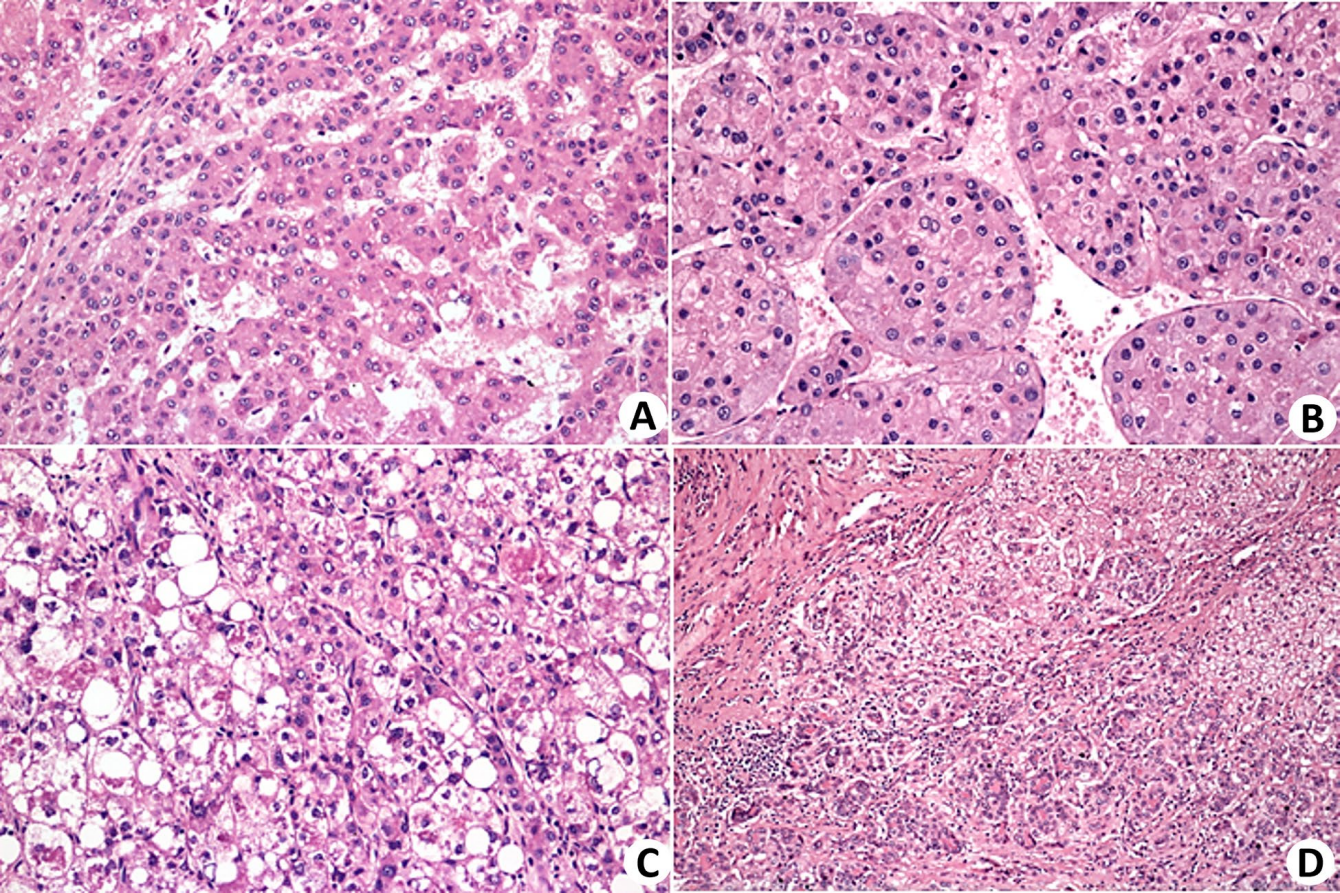
Histopathological aspects of HCC. (**A**) Classic trabecular pattern of HCC, histological grade II, composed of cells with moderate atypia, arranged in thin trabeculae (H&E ×100). (**B**) HCC, macrotrabecular subtype. The trabeculae are thick, with more than 10 cells (H&E, ×200). (**C**) Steatohepatitic subtype of HCC. The neoplastic cells exhibited steatosis, ballooning, and Mallory-Denk bodies (H&E, ×100). (**D**) Combined HCC-CCA. Biphenotypic neoplasia with areas of hepatocellular differentiation alongside areas of cholangiocellular differentiation (H&E ×100).

In six patients, the tumor was solitary, while in three patients, three tumors were identified each. In patients with multiple tumors, evaluations were based on the largest tumor. The size of the tumors ranged from 2 to 5.2 cm (Table 1). Eight out of nine tumors were HCC, one of which was a combined hepatocellular-cholangiocarcinoma (cHCC-CC) tumor (Table 2 and Fig. 1). Six of the HCCs were of the classic conventional type, with a trabecular architecture containing solid areas and some pseudoacinar arrangements. One patient was classified as having the steatohepatitic subtype of HCC, and the other was classified as having the macrotrabecular subtype. The degree of histological differentiation, following WHO recommendations, was considered the least differentiated area. Six tumors were classified as moderately differentiated, and two were classified as poorly differentiated. Vascular infiltration in small caliber venous vessels inside the tumors or at their periphery was observed in five patients.

**Table 2:** Morphological analyses of tumors.

Case	Histological type	Histological grade	Vascular invasion	Hep-Par	Arginase	Glutamine syntase	pCEA	CK7	CK19	EpCAM	CD56	GLI1
HCC5	Conventional HCC	3	0	+	+	+	+	0	0	0	0	ND
HCC6	Macrotrabecular HCC	2	1	+	+	0	+	0	0	0	0	ND
HCC7	Conventional HCC	2	1	+	+	+	+	+	0	0	0	ND
HCC8	Steatohepatitic HCC	3	1	+	+	+	+	+	+	0	0	+
HCC13	cHCC-iCCA	Not applied	0	+	+	+	+	+*	+*	0	0	ND
HCC16	Conventional HCC	2	1	+	+	+	+	+	0	0	0	ND
HCC17	Conventional HCC	2	0	+	+	+	+	0	0	0	0	+
HCC18	Conventional HCC	2	0	+	+	+	+	+	+	0	0	+
HCC22	Conventional HCC	3	1	+	+	+	+	+	0	0	0	ND

Conventional HCC: trabecular, pseudoacinar and solid; cHCC-iCCA: Combined hepatocellular carcinoma and intrahepatic cholangiocarcinoma; ND: Not determined.

*CK7 and CK19 were strongly positive only in the cholangiocarcinoma component of the cHCC-iCCA patient. In the other cases, these markers were only focally detected in precursor hepatic cell-like and intermediate-type hepatocytes.

**Figure 2:**
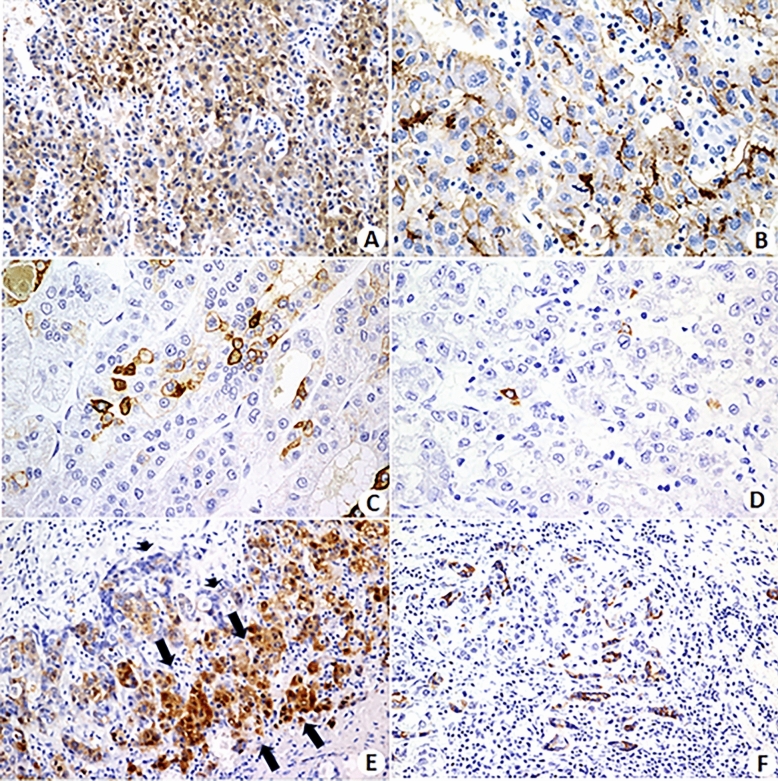
Immunohistochemical staining. (**A**) Immunohistochemistry for arginase shows positive staining in neoplastic hepatocytes. (**B**) Immunohistochemical reaction is positive for pCEA, depicting bile canaliculi formed by neoplastic hepatocytes. (**C**) Cytokeratin 7 is densely positive in small cells with morphological characteristics of hepatic precursor cells and "blushed" in intermediate type hepatocytes. (**D**) A few isolated CK19 positive cells with morphological characteristics of hepatic precursor cells. (**E**) Zone of transition between a hepatocellular carcinoma (HCC) and intrahepatic cholangiocarcinoma (iCCA) in the case of combined HCC-iCCA. Neoplastic hepatocytes are positive for Hep-Par, a marker of hepatocytes (arrows), whereas the cholangiolar component of the tumor is negative. (**F**) Immunostaining for CK7 shows positivity in the biliary component of a combined HCC-CCA tumor. (Magnifications—**A**: ×100; **B**: ×400; **C**: ×200; **D**: ×200, **E**: ×100, and **F**: ×40).

Conventional markers of hepatocellular differentiation were positive in all patients (Table 2 and Fig. 2). Immunohistochemistry was performed using the following antibodies: anti-arginase, anti-polyclonal carcinoembryonic antigen (pCEA, for visualization of bile canaliculi), anti-glutamine synthetase and anti-hepatocyte (Hep-Par). Markers of hepatic precursor cells (cytokeratin 7 [CK7], cytokeratin 19 [CK19], epithelial cell adhesion molecule [EpCAM], and CD56) were also used (Table 2 and Fig. 2). These markers were negative in most patients, and when positive, they were detected in small, isolated foci (CK7 in 5 patients and CK19 in two patients; EpCAM or CD56 proteins were not expressed in any patient). On the other hand, CK7 and CK19 strongly marked the tubular components of cholangiocarcinoma in the case of combined cHCC-CC tumor.

### Identification of HH pathway components in HCC patients

To evaluate the molecular pathways of HCC tumors, in silico analysis of HCC tumors from the TCGA database was used to explore HCC transcriptomics. After processing the HCC-TCGA matrix, the weighted gene coexpression network analysis (WGCNA) package was applied to explore the relationships between selected genes and clinical phenotypes. According to the WGCNA protocol, the Pearson correlation coefficient was used to cluster the samples. To confirm that the network was scale-free, a soft-thresholding power of β = 9 (scale-free R^2^ = 0.9) (Supplementary Figs. 1A and 1B) was applied. A total of six modules were found, and a module-trait relationship heatmap was generated to examine the relationships between the modules and tumor clinical stages (Supplementary Fig. 1C). Only the blue and turquoise modules showed significant relationships (*P* < 0.05) with HCC stage (I, II, III, and IV). These two modules demonstrated the greatest correlations with stages I and III. Thus, these modules were highlighted as clinically important and were subjected to enrichment analysis. The gray module was discarded from the analysis because it represents genes that were not coexpressed.

Gene Ontology (GO) analysis of the blue and turquoise modules revealed biological processes, while enriched pathways were defined through Kyoto Encyclopedia of Genes and Genomes (KEGG) (Supplementary Figs. 2A and 2B). Specifically, the biological process group of genes belonging to the turquoise module (MEturquoise) was mainly associated with cell‒cell signaling and adhesion, extracellular matrix organization, cell proliferation, the cell cycle and cell division. In the blue module (MEblue), the biological processes involved in angiogenesis, blood coagulation, cell adhesion, inflammatory response, cell proliferation, apoptotic process and cell migration are highlighted. In addition, KEGG enrichment analysis was conducted for both modules, and p53 signaling, cell cycle, cellular senescence and ether lipid metabolism were identified as relevant pathways. The blue module was enriched in many cancer-related pathways, such as the TGF-β, Wnt, PI3K-Akt and MAPK signaling pathways; proteoglycans in cancer; cortisol synthesis; and cytokine‒cytokine receptor interaction. These results show that the turquoise and blue modules are closely linked to tumorigenesis and progression.

After screening MEblue and MEturquoise as clinically significant modules, a search for genes related to the HH pathway and their correlations was performed. To measure the importance of the network components, the module connectivity was calculated for all genes through the absolute value of the module membership and the absolute value of gene significance. Generally, gene hubs display high values of module membership and gene significance. *GLI1* and *KIF7* were detected in MEturquoise. In MEblue, *GLI2, GLI3, GAS1*, and *PTCH1* were found to be coexpressed genes. As tumor stage III showed a greater positive correlation with the highlighted modules, the analysis focused on the gene significance and module membership of these stages. As shown in Supplementary Figs. 3A and 3B, the genes with greater numbers of module memberships in the MEturquoise module were more significantly differentially expressed than in the blue module, and they were strongly positively correlated with each other.

Although *GLI1, KIF7, GLI2, GLI3, GAS1,* and *PTCH1* are not hubs of their respective modules, most genes have high connectivity values (module membership > 0.7), apart from *KIF7* and *PTCH1*. These results indicate that these genes are strongly correlated with each other and with the other genes that make up their respective modules.

To identify whether the genes in the coexpression network have prognostic value, overall survival analyses of *GLI1, KIF7, GLI2, GLI3, GAS1*, and *PTCH1* were performed by Kaplan–Meier plotting (Supplementary Figs. 4A–F). In this analysis, a higher expression level of *GLI1* was significantly associated with worse overall survival among HCC patients. The other genes showed a pattern similar to that of *GLI1*; unfortunately, the log-rank p values were not significant for the overall survival curves. Disease-free survival (DFS) analysis revealed that low expression of *GLI3* and *KIF7* was strongly associated with poor prognosis (*P* < 0.05) (Supplementary Figs. 5A–F).

The number of transcripts per million in tumor and normal tissues was compared (Supplementary Figs. 4G–L). The gene expression levels of *GLI1, GLI4* and *KIF7* were significantly greater in tissues from at least stage III tumors than in normal tissues. There were no significant differences in *GLI3* or *PTCH1* expression between normal and tumor tissues.

**Figure 3:**
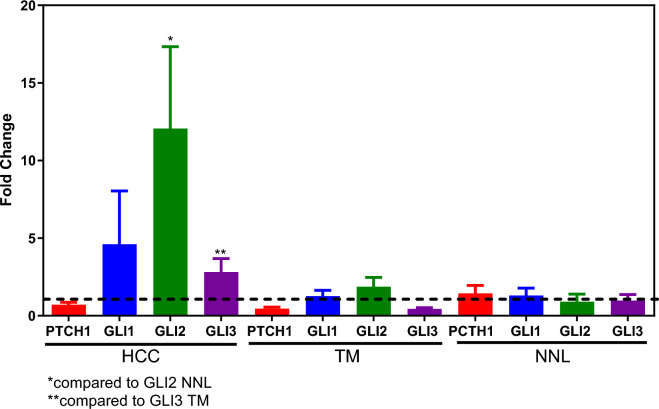
Fold changes in *PTCH1*, *GLI1*, *GLI2* and *GLI3* expression in patients with HCC, including tumors, tumor lateral margin (TM) at the interface with the nonneoplastic liver, and distant nonneoplastic liver tissue (NNL) far from the tumor. The data are shown as the means ± S.E.M.s of nine patients.

Validation of the results observed in the in silico study was subsequently carried out in our nine HCC patients. To analyze whether HH signaling is present in HCC samples, first, the expression of HH signaling genes (*PTCH1, GLI1, GLI2* and *GLI3*) was examined in tissue samples of HCC, TM and NNL by qPCR (Fig. 3 and Supplementary Table 1). The *GLI1* (RQ = 4.60), *GLI2* (RQ = 12.07), and *GLI3* (RQ = 2.81) genes were overexpressed in our HCC samples. The mRNA levels of *PTCH1* (RQ = 0.45) and *GLI3* (RQ = 0.44) were downregulated in the TM. In the NNL, the transcript levels of all the genes investigated were similar to those observed in nonneoplastic liver tissue (a calibrator). Greater levels of *GLI2* transcripts were detected in HCC tissues than in NNL tissues, and greater expression of the *GLI3* gene was detected in HCC tissues than in TM tissues.

**Figure 4:**
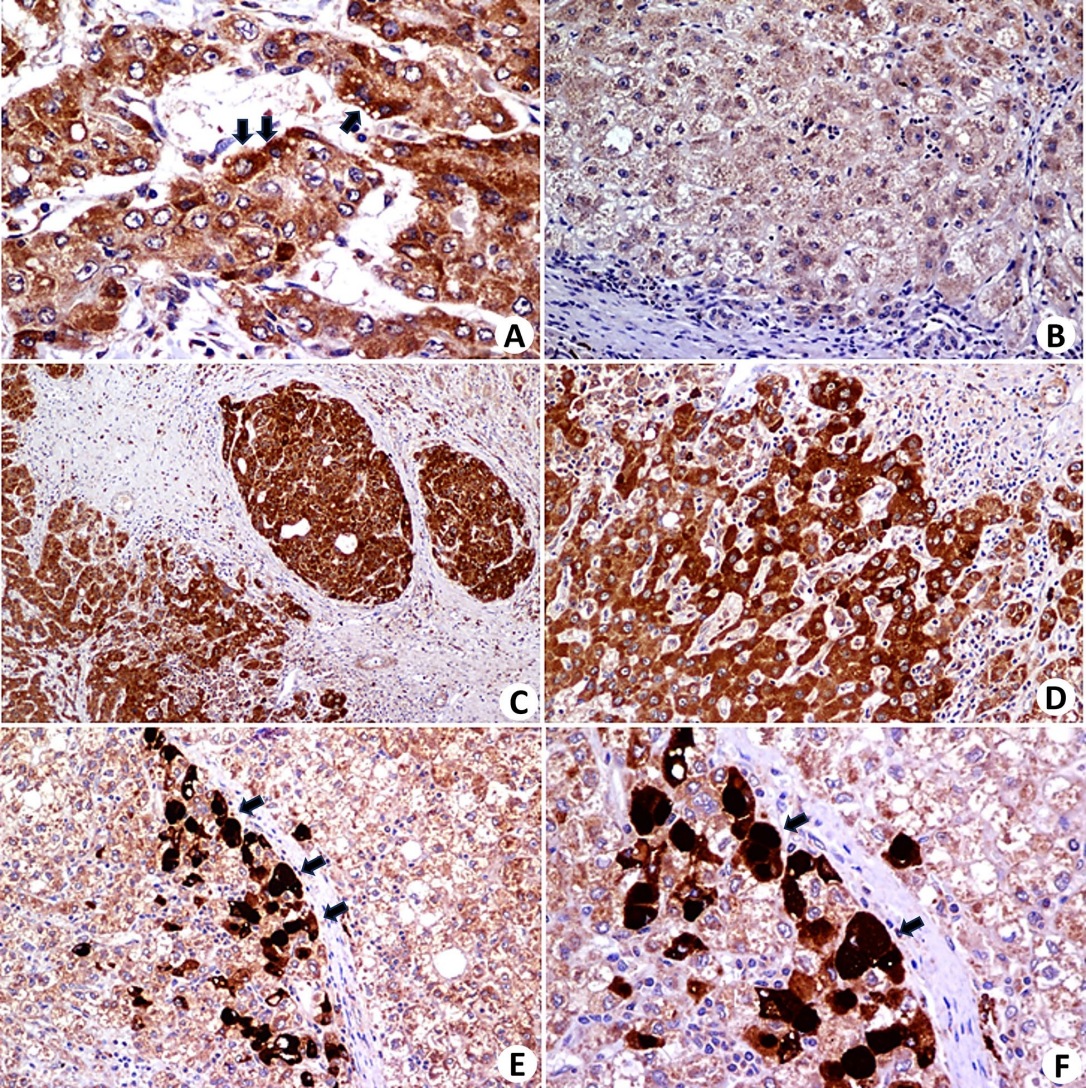
Immunohistochemistry with an anti-GLI1 monoclonal antibody. (**A**,**C**,**D**,**E**, and **F**) show the protein expression in HCC cells from three patients. The intensity of the expression was heterogeneous, with very dense areas in some cells (Figures **E** and **F**). The expression appears to be more intense in the interface zone of the neoplasm with the stroma in (**A**,**E**, and **F**) (arrows). Figure (**B**) shows a nonneoplastic hepatic parenchymal nodule without protein expression or with very weak expression (Magnifications—**A**: ×400; **B**: ×100; **C**: ×40; **D**: ×100, **E**: ×40, and **F**: ×200).

Next, immunohistochemistry was performed using an anti-GLI1 antibody (Table 2 and Fig. 4). GLI1 expression was positive in all three patients in which it was tested. The staining was cytoplasmic and stronger in some cells. In one of the cases, there were foci of intense staining. In comparison to the liver outside the tumor, the difference is evident.

### In vitro chemosensitivity of HCC patients

The chemosensitivity of all nine HCC samples and the HepG2 liver cancer cell line to a panel of 18 drugs (methotrexate [MTX], dacarbazine [DTIC], etoposide [VP-16], vinblastine [VBT], melphalan [MPL], 7-ethyl10-hydroxycamptothecin [SN-38, the active metabolite of irinotecan], cisplatin [CDDP], cyclophosphamide [CP], chlorambucil [CRB], paclitaxel [PTX], fludarabine [FDB], bendamustine [SDX-105], sunitinib [SNT], cytarabine [ara-C], doxorubicin [DOX], mitoxantrone [MIT], oxaliplatin [OXA], and 5-fluorouracil [5-FU]) was evaluated after 72 h of incubation using the Alamar blue method. All drugs were tested at a concentration of 25 μg/mL, and an inhibition rate greater than 50% was defined as the sensitivity value for drug evaluation.

The cytotoxicity results are described in Table 3 and Supplementary Figs. 6–15. SNT inhibited cell viability by more than 50% in six of our HCC samples. At least three HCC samples were sensitive to ara-C, DOX, FDB, SN-38, OXA and VBT. At least two HCC samples were sensitive to SDX-105, MPL, and 5-FU, and at least one HCC sample was sensitive to CRB, VP-16 and MIT. No chemosensitivity to any drug was detected in three HCC samples. MTX, DTIC, CDDP, and PTX only demonstrated cytotoxicity to HepG2 cells. The other eight chemotherapeutic agents (DOX, VP-16, FDB, SN-38, MIT, OXA, SNT and 5-FU) also demonstrated cytotoxicity to HepG2 cells.

**Table 3:** Chemosensitivity of tumors.

Case	Sensitive drugs
HCC5	Fludarabine, bendamustine, sunitinib, oxaliplatin and 5-fluorouracil
HCC6	Sunitinib and doxorubicin
HCC7	None
HCC8	Sunitinib, doxorubicin and oxaliplatin
HCC13	Fludarabine, sunitinib and cytarabine
HCC16	Vinblastine, 7-ethyl10-hydroxycamptothecin, bendamustine, cytarabine, doxorubicin, mitoxantrone and 5-fluorouracil
HCC17	None
HCC18	None
HCC22	Etoposide, vinblastine, melphalan, 7-ethyl10-hydroxycamptothecin, chlorambucil, fludarabine, sunitinib, cytarabine and oxaliplatin

Cell viability was determined by the Alamar blue method after 72 h of incubation with a panel of 18 drugs at a concentration of 25 μg/mL. An inhibition rate greater than 50% was defined as the sensitivity value for drug evaluation.

## Discussion

In this study, we characterized a series of nine patients with HCC, established a morphological and molecular baseline using traditional HCC immunomarkers and employed in silico analysis of HCC tumors from the TCGA. Our research revealed that all nine HCC samples exhibited positive staining for traditional HCC biomarkers despite explantation. This finding corroborates that HCC derived from explants can be a helpful sample for studying primary liver cancer^19,20^. The HH genes were strongly associated with HCC gene networks, with the *GLI1*, *GLI2*, and *GLI3* genes being overexpressed in the samples. Additionally, we tested a chemosensitivity panel analysis using these primary HCC samples, noting a particular sensitivity to sunitinib in most cases.

LT is a well-established treatment for HCC, as this therapeutic approach offers two benefits: radical oncological resection and resolution of liver dysfunction^21^. The recurrence rate in the liver post-LT can reach 15%, depending on the criteria used to indicate eligibility for surgical treatment^22,23^. Studies have discussed the necessity of developing strategies to evaluate explanted liver samples, thus better characterizing the risk of recurrence and the use of complementary therapies^20^. Tumor size, grade, and the number of nodules have all been used as predictors of recurrence-free survival^24^.

Herein, we delineated a set of HCC immunomarkers for liver cancer patients. Biomarkers have been investigated in HCC for prognostication and therapeutic decisions^25,26^. In this study, conventional markers of hepatocellular differentiation (Hep Par-1, arginase, pCEA, and glutamine synthetase)^26–29^ were detected in all patients. In particular, arginase represents a sensitive marker of HCC, even in poorly differentiated HCC and scirrhous HCC, but is not considered a uniquely promising prognostic biomarker^24^. In addition, the positivity of hepatic precursor cell biomarkers (CK19, CK7, EpCAM, and CD56) tends to decrease in poorly differentiated tumors^3,25,27,29,30^.

To address the complexity and intriguing molecular landscape of HCC, an in silico transcriptomic analysis of HCC from TCGA was performed. Analysis demonstrated that HH signaling genes (e.g., *PTCH1, GLI1, GLI2, GLI3,* and *GLI4*) are biomarkers due to their greater connectivity with the HCC gene network. The HH pathway is involved in embryonic liver development, and its reactivation has been linked to the promotion of proliferation, viability, migration, invasion, and maintenance of metastasis in HCC^31^. Deregulation of pathways can participate in the pathogenesis of HCC via both canonical and/or noncanonical routes^32–36^.

In the canonical pathway, one of the HH ligands (Sonic, Indian, or Desert) interacts with PTCH1, resulting in the accumulation of SMO in the primary cilium^37,38^. HH signals are transduced to the cytoplasm, and transcription factors of glioma-associated oncogene homolog (GLI) migrate to the nucleus and activate target genes involved in proliferation and survival^39^. Activation of the HH pathway plays an important role in cellular proliferation, differentiation, invasion, and cancer stem cell maintenance^31,40–43^.

Herein, further validation in HCC samples identified *GLI1* as a key gene. This molecule was present in the most biologically relevant coexpression module (MEturquoise) with a higher module membership value (> 0.7) and correlated with overall survival. GLI1 is a key effector in the HH pathway^37,38^, and its overexpression confirmed that the HH pathway is activated in HCC, contributing to drug resistance by maintaining stem-like properties, the onset of epithelial–mesenchymal transition and the induction of ABC transporters in HCC^16^. In addition, this study highlights its significance as a potential therapeutic target.

HCC treatment is still a challenge due to the lack of therapeutic options for advanced disease, especially with conventional cytotoxic drugs that are usually ineffective^44^. In this context, primary cell culture approaches provide a reasonable accurate representation of the in vivo state for chemosensitivity testing^45^. Therefore, several primary HCC cell culture techniques have been developed to test drug sensitivity in vitro^44,46,47^.

In this study, a chemosensitivity analysis was performed in primary HCC using a selected panel of 18 drugs exploiting their distinct mechanisms of action, ranging from DNA damage to mitotic inhibition or previous scientific evidence of the effects of these chemotherapeutic agents on the HH cascade in HCC. Our HCC primary cells were most sensitive to sunitinib, a tyrosine kinase inhibitor related to sorafenib, which is an approved drug for the treatment of HCC^48^. Sutinib has shown promising results in patients with advanced HCC and mild to moderately impaired liver function^49,50^. Its mechanisms are related to targeting vascular endothelial growth factor receptor (VEGFR) and platelet-derived growth factor receptor (PDGFR), which play essential roles in the growth and spread of cancer^51^, including HCC^49^. Sunitinib has also been reported to synergize with radiofrequency ablation for treating HCC^52^.

The results of this study have limitations that need to be further considered. Our sample size was limited, and our findings do not represent the histological heterogeneity of HCC. In addition, some clinical and pathological aspects were not adjusted, for example, the time of transplant. In chemosensitivity assays, primary cells have a limited survival period in culture, as do those in artificial tumor microenvironments.

In conclusion, this study comprehensively analyzed traditional HCC markers in nine patients with liver cancer. Transcriptomic analysis of the TCGA database provides valuable insights into the molecular landscape of HCC. By further validating these findings, we identified GLI1, GLI2, and GLI3 as essential molecules in HCC, highlighting the significance of the HH pathway and opening discussions for focused treatments in HCC. In addition, a chemosensitivity panel using primary HCC cells corroborated the use of sunitinib as a potential drug for HCC treatment.

## Materials and methods

### Clinical and morphological characterization

Between January 2015 and December 2016, this study evaluated nine cases of HCC, including tumor (T), lateral margin (TM) at the interface with the nonneoplastic liver, and liver distant from the neoplasm (NNL) samples. Six of the cases involved hepatic explants, and three involved hepatic segmentectomy for tumor resection.

The cases were selected so that we could study the morphological, molecular and chemosensitivity aspects in pairs. This study was carried out with patients from Portugues Hospital (Salvador, Bahia, Brazil), and the Research Ethics Committee of the Oswaldo Cruz Foundation (Salvador, Bahia, Brazil) approved the protocol (CAAE: 18417813.5.0000.0040). This study was conducted according to the Declaration of Helsinki, and all patients provided informed written consent for the purpose of the research.

Under sterile conditions, the samples were immediately transferred from the operating room to the laboratory for sectioning by an experienced pathologist (L.A.R.F.). Three fragments were harvested from each tumor: one was stored in RNAlater (Invitrogen Corporation, USA) for 24 h and frozen in freezer (− 80 °C), the other was fixed in 10% neutral buffered formalin for histological processing, and the other was used to obtain primary HCC cells. The TM and NNLT fragments were also preserved in RNAlater (Invitrogen Corporation, USA) for further analysis.

After fixation in 10% neutral buffered formalin, the tumor and nonneoplastic liver samples were dehydrated in alcohol, embedded in paraffin, after xylene baths, and then sectioned to a thickness of 3–5 microns. These sections were stained with hematoxylin and eosin for histological evaluation following WHO recommendations for the classification of histological types and subtypes, as well as histological grading^53^.

Immunohistochemical evaluation of the tumors was performed using the following conventional markers of hepatocellular differentiation: Hep Par-1, arginase, pCEA, and glutamine synthetase. In addition to these markers, CK7, CK19, EpCAM, and CD56 were used to evaluate the presence of hepatic precursor cells in the neoplasms. The expression of GLI1 was evaluated in three patients using a polyclonal antibody. Supplementary Table 2 shows the antibodies used and their suppliers.

The histological sections were deparaffinized in xylene and then rehydrated with alcohol. To expose the antigenic epitopes, the sections were subjected to antigen retrieval under moist heat for 45 min. Subsequently, endogenous peroxidase blocking (Peroxidase Blocking Solution™, Dako, Carpinteria, USA) and tissue protein blocking (Protein Blocking Solution™, Dako) were carried out. The slides were incubated with primary antibodies overnight at 4 °C, followed by the application of HRP Link and HRP Enzyme reagents (Advance™, Dako). The reactions were visualized using 3,3'-diaminobenzidine (Dako) and counterstained with Harris hematoxylin. For negative controls, in each reaction, the specific primary antibodies were replaced with an unrelated IgG of the same isotype.

### In silico study

RNA-seq data for human HCC samples were obtained from the TCGA database (portal.gdc.cancer.gov/projects/TCGA-LIHC). The gene expression data and corresponding clinical information were downloaded from the TCGAbiolinks package (0.18129/B9.bioc. TCGAbiolinks), version 2.1.0 (371 HCC patients and 50 healthy tissues). The edgeR package (version 3.10.5) was used to filter genes with low counts^54^. All genes with a CPM (counts per million) > one were included for further analysis. Subsequently, a total of 27,644 genes were processed using variance analysis, and the top 25% of the most variant genes (6,911) were selected for coexpression network construction.

The WGCNA (version 1.70-3)^55^ package in R was used to construct a gene coexpression network with 6,911 genes. Coexpression analysis was performed for paired genes using a Pearson correlation matrix. Next, the weighted adjacency matrix was constructed using the power function as follows:

$${a}_{ij}= {s}_{ij}^{\beta }$$

In this formula, ${s}_{ij}$ represents Pearson's correlation between gene *i* and gene* j*. In addition, β is a soft thresholding approach that leads to a weighted gene coexpression network with a scale-free trait. These values should be chosen in a way that emphasizes strong correlations between genes and penalizes weak correlations. Next, the TOM matrix is calculated from the adjacency matrix to delineate the similarity in nodes, correlating the weighted correlation between two nodes and the other nodes. Finally, to identify genes with absolute highs and cluster them into modules for further analysis, average linkage hierarchical clustering was performed based on TOM-based dissimilarity. The minimum number of genes per module was set to 60.

To select the key modules for the analysis, the correlations between module eigengenes, which is the first principal component analysis of the gene module, and clinical traits were assessed. Module significance is defined by the average gene significance for all genes in each module. Modules with higher module significance are biologically relevant for a given condition. Gene significance is reached by a linear relationship between gene expression and clinical traits. In addition, the module membership was measured for all genes and represented its connectivity. All analyses were conducted using R (version 3.6.3)^56^.

GO and KEGG pathway enrichment analyses of the WGCNA modules were performed using the DAVID platform (https://david-d.ncifcrf.gov). To select the most significant GO and KEGG terms, “adjusted” *P* < 0.01 was used.

### Molecular biology

The samples were homogenized (L-Beader, Loccus Biotecnology). Total RNA was extracted using microcolumns (RNeasy Plus Mini Kit, QIAGEN, Tokyo, Japan). RNA purity was evaluated by spectrophotometry (NanoDrop™, Thermo Scientific, Wilmington, USA), while quantification was determined by fluorimetry (QuBit™, Life Technologies, USA). Genomic DNA was eliminated using DNAse I enzyme (Invitrogen Corporation, Carlsbad, CA). For reverse transcription, RNA was processed using the SuperScript® VILO™ cDNA Synthesis Kit (Invitrogen Corporation, Carlsbad, CA). The experiments were performed under conditions free of DNAse/RNAse. TaqMan gene inventoried assays for *GLI1* (Hs01110755_m1), *GLI2* (Hs01119974_m1), *GLI3* (Hs00609233_m1) and *PTCH1* (Hs00181117_m1) were used for the qPCR study. After evaluating a total of six (*18S, ACTB, B2M, GAPDH, HPRT1,* and *UBC*) reference gene candidates, *ACTB* (Hs01060665_g1) and *GAPDH* (Hs02758991_g1) were selected for gene expression normalization. A nonneoplastic liver sample was used as a normal control for calibration purposes.

All reactions were developed using an ABI ViiA7 Fast Real-Time PCR System (Applied Biosystems™, Foster City, CA) in a 96-well plate at a total volume of 20 μL, with 8 μL of sample cDNA (20 ng/μL), 1 μL of assay (Applied Biosystems™, Foster City, CA), 10 μL of TaqMan PCR Master Mix (Applied Biosystems™, Foster City, CA) and 1 μL of RNAse-free water. The amplification protocol consisted of an initial cycle at 50 °C (2 min) and 95 °C (10 min) followed by 40 cycles (95 °C) for 15 s and 60 °C (1 min).

After amplification and dissociation, relative quantification (RQ) values were obtained using Gene Expression Suite™ v.1.0.3 software (Applied Biosystems™, Foster City, CA) following a comparative method for Cq (2^−ΔΔCQ^)^57^.

### In vitro chemosensitivity assay

One fragment from each tumor was preserved in RPMI-1640 medium (Gibco-BRL, Gaithersburg, MD, USA) supplemented with 10% fetal bovine serum (Life, Carlsbad, CA, USA), 2 mM L-glutamine (Vetec Química Fina, Duque de Caxias, RJ, Brazil) and 50 μg/mL gentamycin (Life, Carlsbad, CA, USA). Tumor fragments were cut with scissors, washed with medium and treated enzymatically for 30 min at 37 °C with 0.5 mg/mL protease, 0.2 mg/mL collagenase type I and 0.2 mg/mL DNase (all from Sigma‒Aldrich Co., Saint Louis, MO, USA). A nylon membrane (100 μm) (Falcon 2350, Becton Dickinson, NJ, USA) was used for filtration. The tumor cell pellet was resuspended in the same complete medium, and the viable cell count was determined by the trypan blue dye (Gibco-BRL, Gaithersburg, MD, USA) exclusion method.

To compare the results for primary cells, the well-characterized human liver cancer cell line HepG2 was obtained from the American Type Culture Collection (ATCC, Manassas, VA, USA) and cultured in flasks. HepG2 cells were subcultured every 3–4 days to maintain exponential growth. Treatment with 0.25% trypsin EDTA solution (Gibco-BRL, Gaithersburg, MD, USA) was used to obtain the cell suspension. Additionally, a mycoplasma stain kit (Sigma‒Aldrich Co., Saint Louis, MO, USA) was used to confirm that the cells were free from contamination.

Chemosensitivity was evaluated in vitro using the Alamar blue method^58^. The cells were seeded in 96-well plates for all experiments (100 μL of a suspension of cells at 5 × 10^5^ cells/mL for primary culture or 7 × 10^4^ cells/mL for HepG2 cells in each well). After 24 h, the drugs (all at a concentration of 25 μg/mL) were added to each well and incubated for 72 h. All drugs were dissolved in DMSO at a stock concentration of 5 mg/mL and stored at − 20 °C.

Four hours before the end of incubation, 20 μL of a stock solution (0.312 mg/mL) of alamar blue (resazurin, Sigma‒Aldrich) was added to each well. The absorbance at 570 nm and 600 nm was measured using a SpectraMax 190 Microplate Reader (Molecular Devices, Sunnyvale, CA, USA). The inhibition rate (IR) was calculated using the following formula: IR (%) = (1 − T/C) × 100, where T and C represent the absorbance of the drug-treated and control wells, respectively. A mean control tumor absorbance < 0.1 was defined as unsuitable for assessment due to an insufficient number of viable cells as a control, and only samples with a mean control tumor absorbance > 0.1 were accepted. An inhibition rate greater than 50% was defined as the sensitivity value for drug evaluation.

### Statistical analysis

The Kruskal‒Wallis test and Dunn’s posttest were employed to compare three or more groups, and Spearman's rank correlation coefficient was used to evaluate correlations between two variables. The overall survival of HCC patients was calculated using the Kaplan‒Meier method and the Cox regression model, and the log-rank test was used to compare survival curves. DFS was calculated with the survival analysis tool KMplotter (http://kmplot.com/analysis/). The results were considered statistically significant when *P* < 0.05. GraphPad Prism 6.03 was used for statistical analysis (GraphPad Software Inc., San Diego, USA).

### Ethics statement

The Research Ethics Committee of the Oswaldo Cruz Foundation (Salvador, Bahia, Brazil) approved the protocol (CAAE: 18417813.5.0000.0040), which was conducted according to the Declaration of Helsinki. All included patients provided informed written consent for the purpose of the research.

### Supplementary Information

Supplementary Information.

## Data Availability

The datasets used and/or analyzed during the current study are available from the corresponding authors upon reasonable request.
