# Supplementary material for: Hedgehog components are overexpressed in a series of liver cancer cases
Source: Sci Rep. 2024 Aug 22;14:19507. doi: 10.1038/s41598-024-70220-0 (PMC11341691; doi:10.1038/s41598-024-70220-0)
Supplement: Supplementary file 1 — Supplementary Information. [file 41598_2024_70220_MOESM1_ESM.pdf]

Supplementary Material

Hedgehog components are overexpressed in a series of liver cancer cases

Caroline Brandi Schlaepfer Sales[1], Rosane Borges Dias[2,3,4], Ludmila de Faro Valverde[2,5], Larissa M. Bomfim[2], Lais Almeida Silva[2], Nanashara C. de Carvalho[2], Jorge Luiz Andrade Bastos[6], Tatiana Martins Tilli[7,8], Gisele Vieira Rocha[2,9], Milena Botelho Pereira Soares[2,10,*], Luiz Antonio Rodrigues de Freitas[2,6,*], Clarissa A. Gurgel Rocha[2,3,9,*], Daniel P. Bezerra[2,*]

[1]Department of Biomorphology, Institute of Health Sciences, Federal University of Bahia (UFBA), Salvador, Bahia, 40110-902, Brazil.

[2]Gonçalo Moniz Institute, Oswaldo Cruz Foundation (IGM-FIOCRUZ/BA), Salvador, Bahia, 40296-710, Brazil.

[3]Department of Propedeutics, School of Dentistry of the Federal University of Bahia (UFBA), Salvador, Bahia, 40110-909, Brazil.

[4]Department of Biological Sciences, State University of Feira de Santana (UEFS), Feira de Santana, Bahia, 44036-900, Brazil.

[5]Department of Dentistry, Federal University of Sergipe (UFS), Lagarto, Sergipe, 49400-000, Brazil.

[6]Medical School of Bahia, Federal University of Bahia (UFBA), 40110-100, Brazil.

[7]Translational Oncology Platform, Center for Technological Development in Health, Oswaldo Cruz Foundation (FIOCRUZ), Rio de Janeiro, Rio de Janeiro, 21040-900, Brazil.

[8]Laboratory of Cardiovascular Research, Oswaldo Cruz Foundation (FIOCRUZ), Rio de Janeiro, Rio de Janeiro, 21040-900, Brazil.

[9]D'Or Institute for Research and Education (IDOR), São Rafael Hospital Center for Biotechnology and Cell Therapy, Salvador, Bahia, 41253-190, Brazil.

[10]SENAI Institute for Innovation in Advanced Health Systems, SENAI CIMATEC, Salvador, Bahia, 41650-010, Brazil.

**Corresponding authors:*

M.B.P. Soares (e-mail: milena.soares@fiocruz.br), Tel. +55-71-3176-2272; L.A.R. Freitas (e-mail: freitas.luizar@gmail.com), Tel. +55-71-3176-2272; C.A. Gurgel Rocha (e-mail: clarissa.gurgel@fiocruz.br), Tel. +55-71-3176-2289; D.P. Bezerra (e-mail: daniel.bezerra@fiocruz.br), Tel. +55-71-3176-2272.

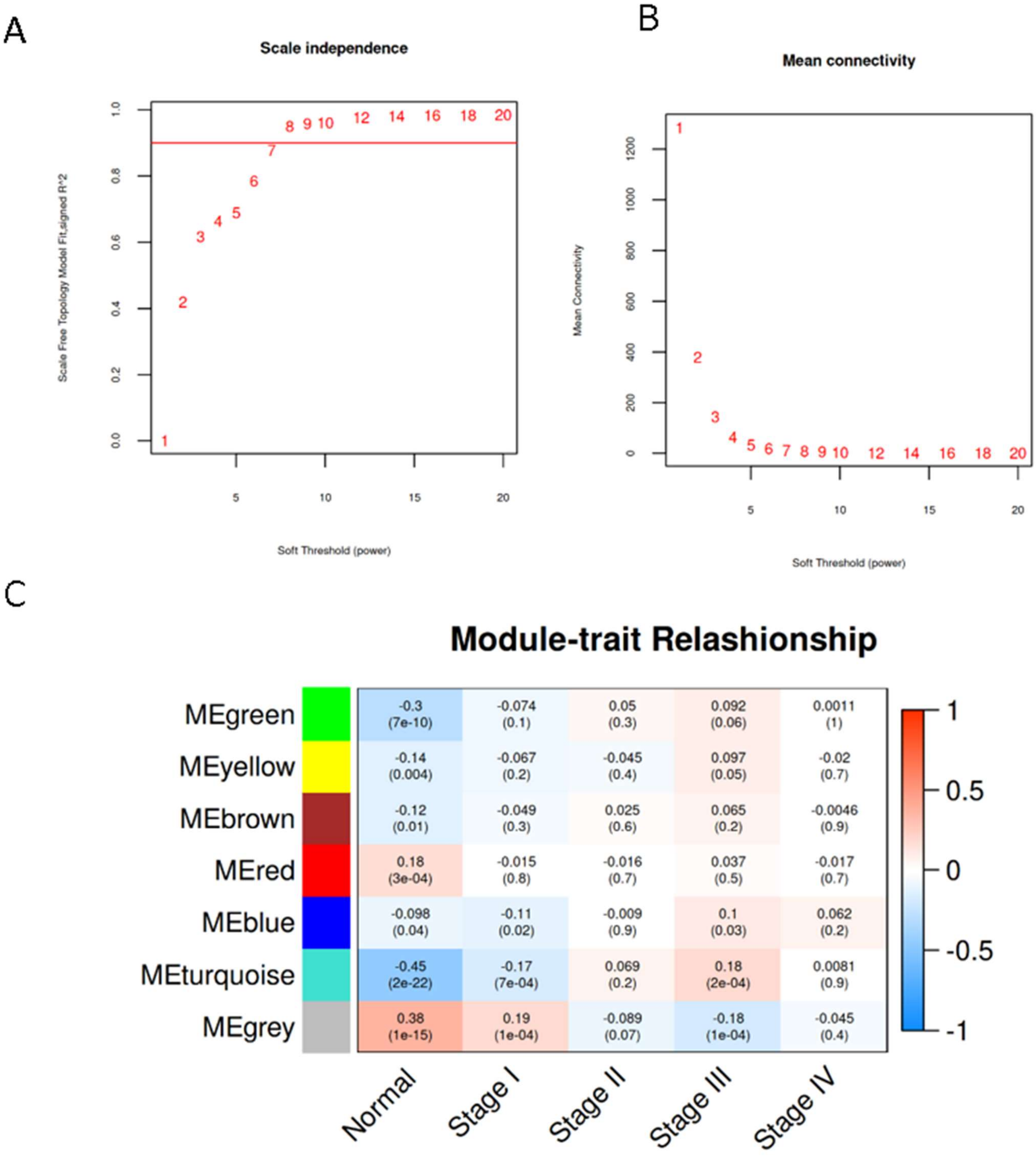

Supplementary Figure 1. Weighted gene coexpression network construction and identification of modules associated with the clinical traits of HCC patients. (**A**) Analysis of the scale-free fit index to select soft-thresholding powers. (**B**) Analysis of the mean connectivity for various thresholding powers. (**C**) Heatmap of the correlation between module features and clinical traits. The numbers in each cell represent the corresponding correlation and *P* value.

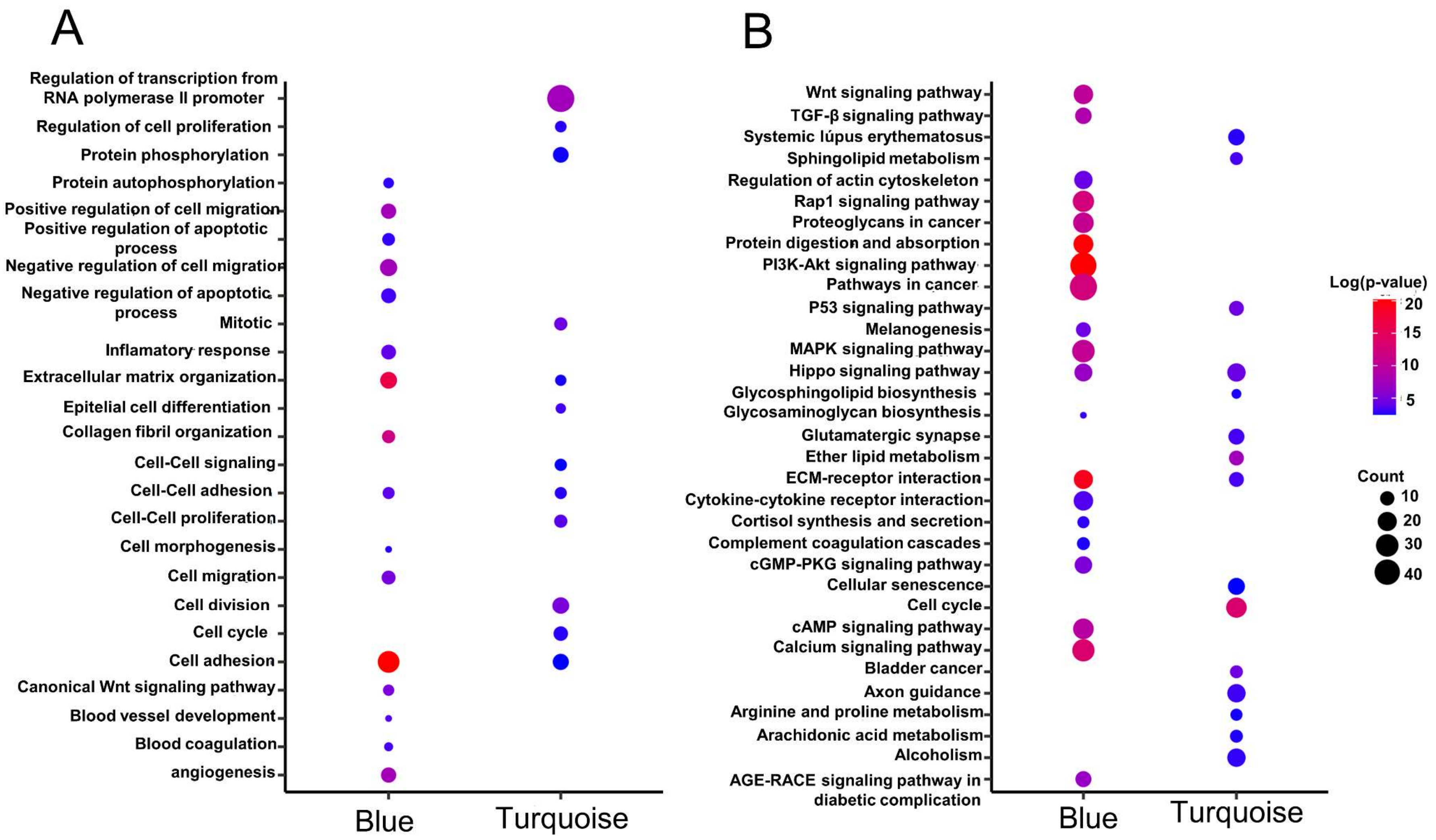

Supplementary Figure 2. Representative results of KEGG pathway and Gene Ontology (GO) enrichment analyses in the turquoise and blue modules. **(A)** GO biological process analysis**. (B)** KEGG pathway analysis.

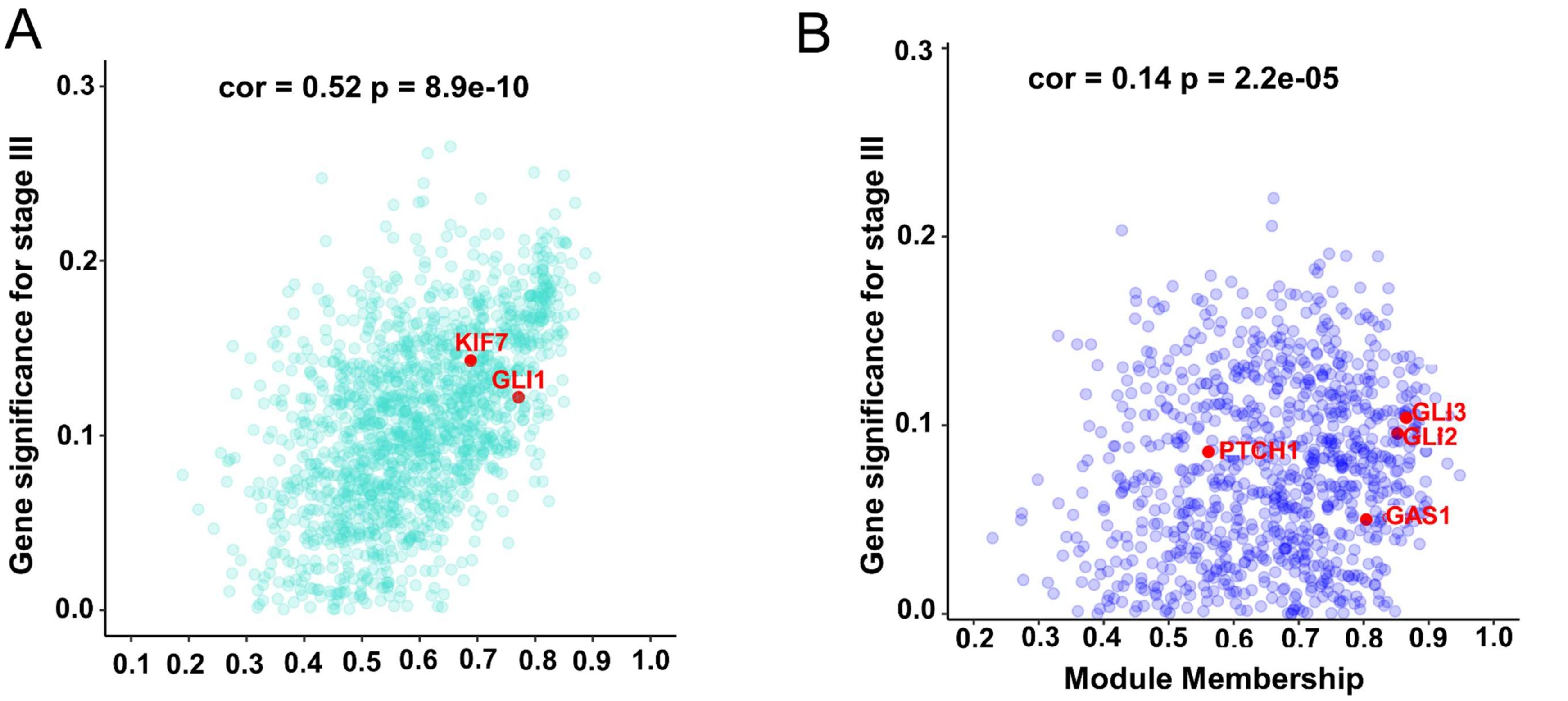

Supplementary Figure 3. Screening of HCC genes related to clinical stage. Scatterplots of absolute gene significance vs module membership for stage III in the turquoise (**A**) and blue (**B**) modules.

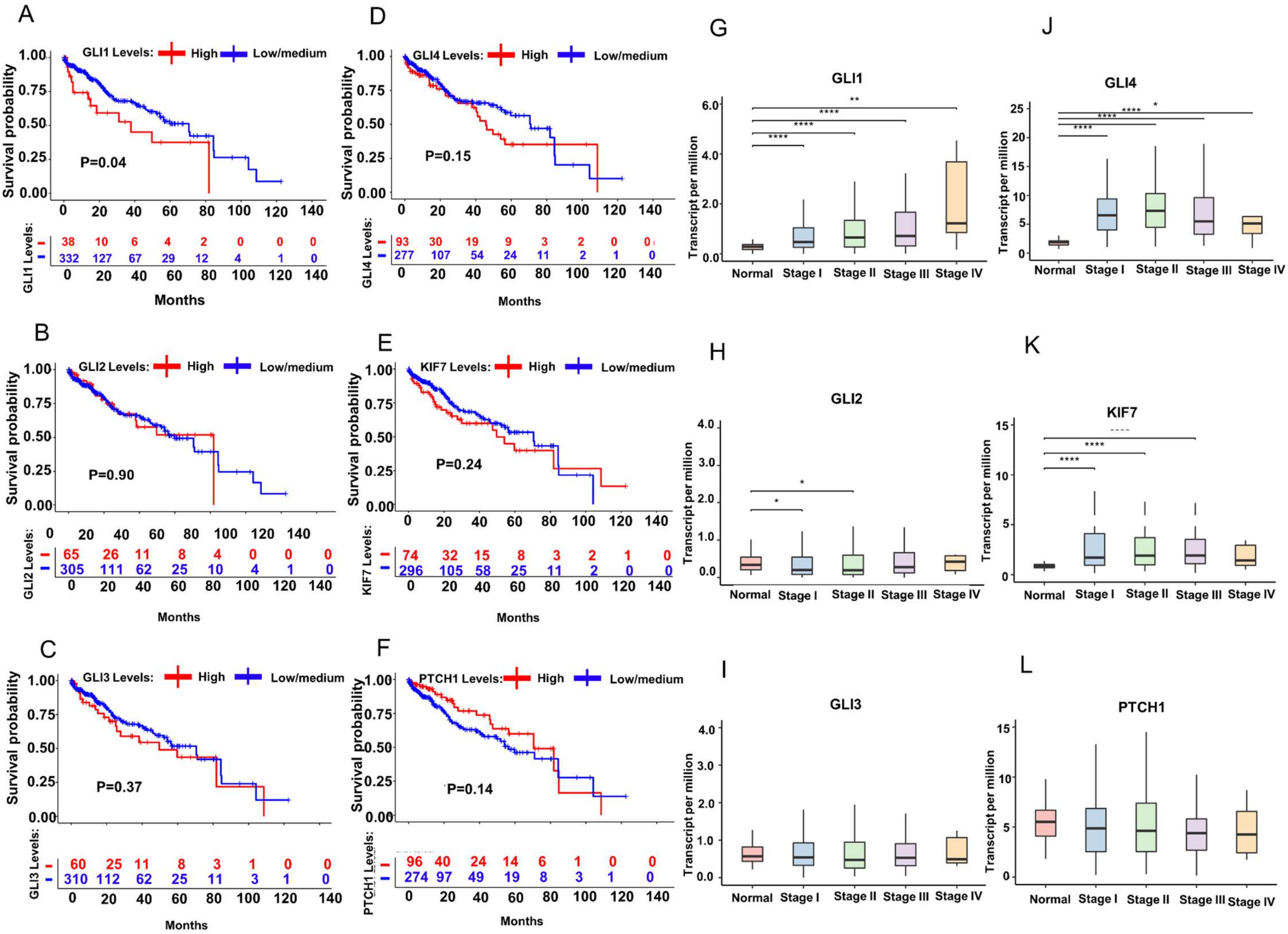

Supplementary Figure 4. Overall survival analysis and expression levels of genes found in coexpression networks. (**A-F**) The overall survival analysis for patients with expression of *GLI1*, *GLI2*, *GLI3*, *GLI4*, *KIF7* and *PTCH1*. (**G-L**) Expression level represented by transcripts per million genes in the HH pathway between normal tissues and tumor stages.

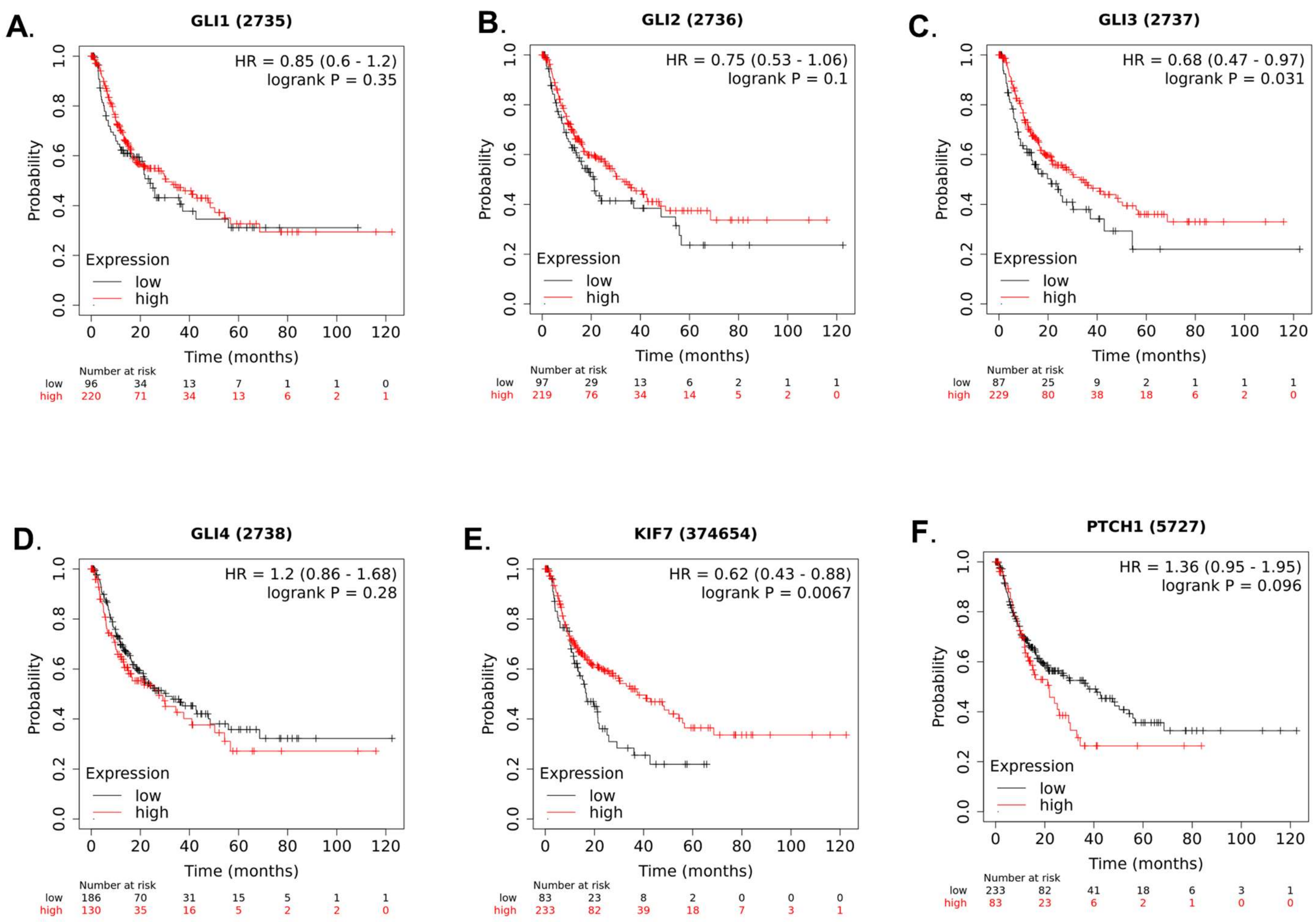

Supplementary Figure 5. Disease-free survival (DFS) analysis and expression levels of genes in the turquoise and blue modules. (**A-F**) DFS analysis for *GLI1*, *GLI2*, *GLI3*, *GLI4*, *KIF7* and *PTCH1*.

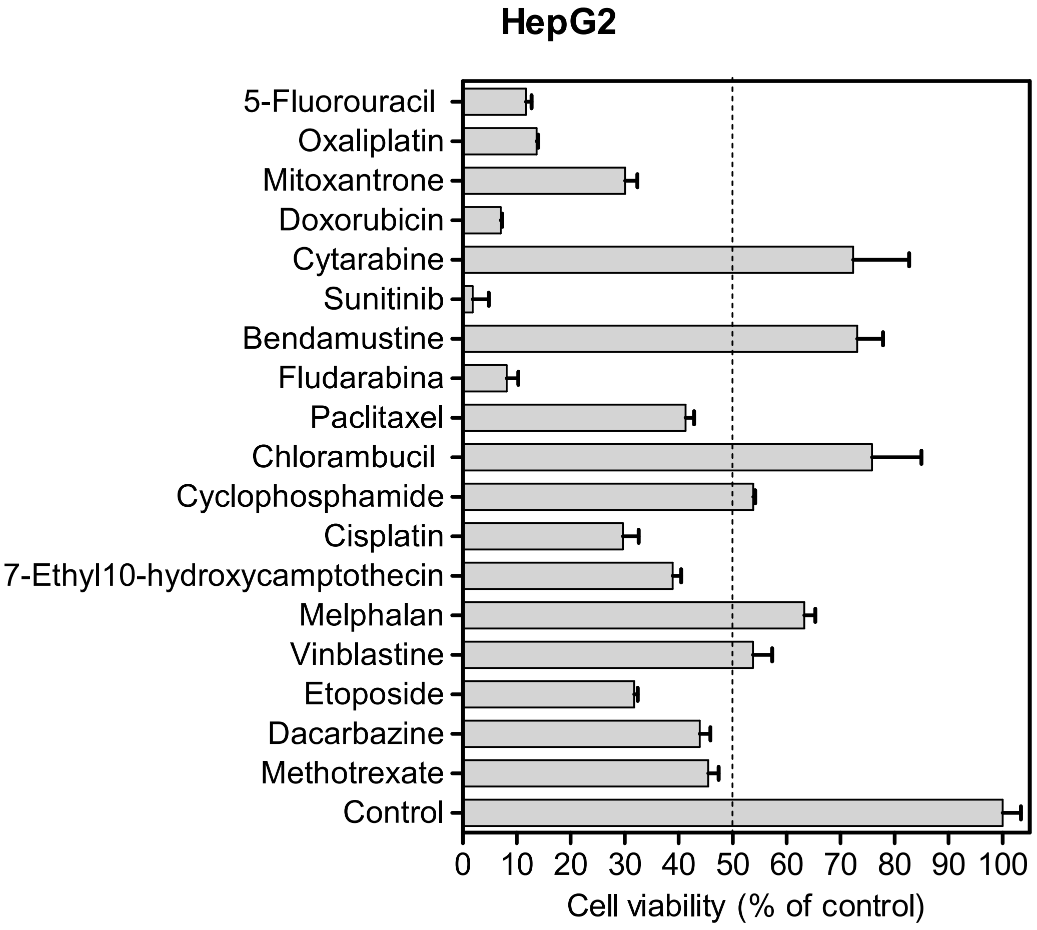

Supplementary Figure 6. Chemosensitivity of HepG2 cells. Cell viability was determined using the alamar blue method after 72 h of incubation with a panel of 18 drugs each at a concentration of 25 μg/mL. An inhibition rate greater than 50% was defined as the sensitivity value for evaluating the drug. The data are shown as the mean ± S.E.M. of three biological replicates performed in duplicate.

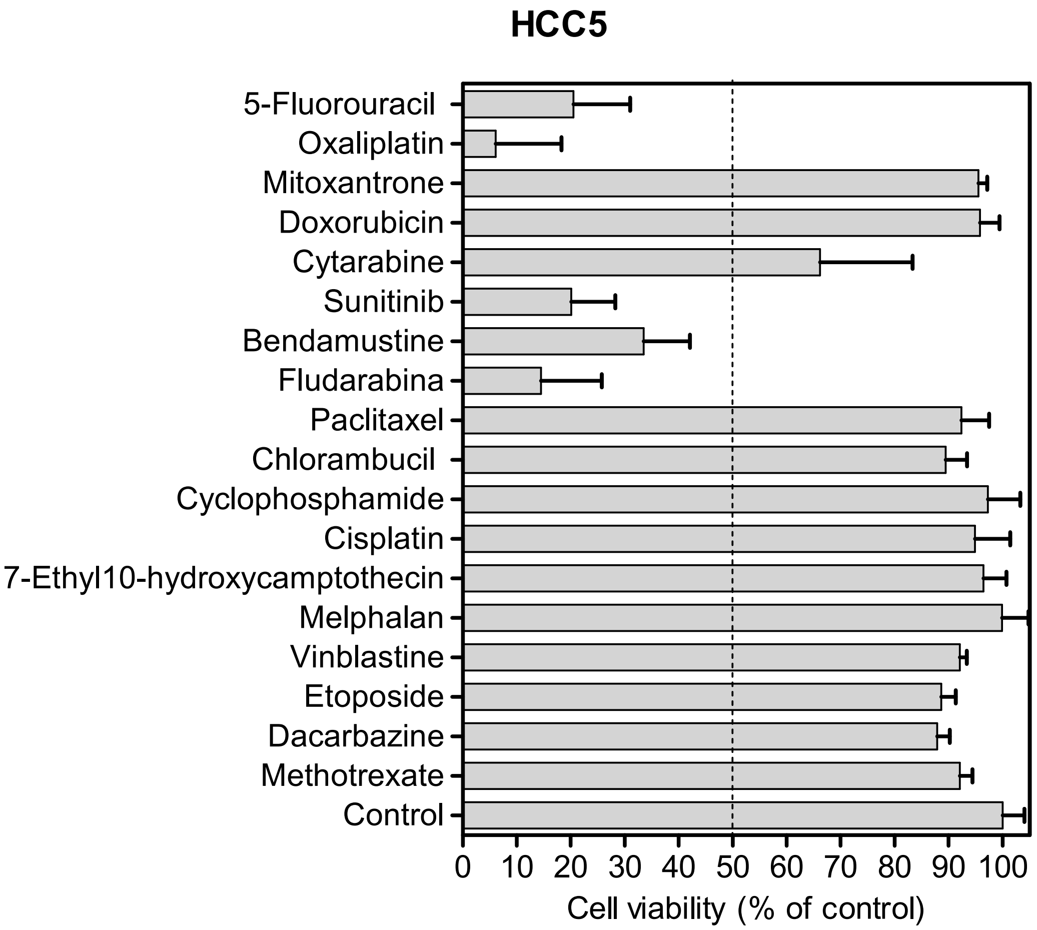

Supplementary Figure 7. Chemosensitivity of the HCC5 liver cancer patient. Cell viability was determined using the alamar blue method after 72 h of incubation with a panel of 18 drugs each at a concentration of 25 μg/mL. An inhibition rate greater than 50% was defined as the sensitivity value for evaluating the drug. The data are shown as the mean ± S.E.M. of eight replicates.

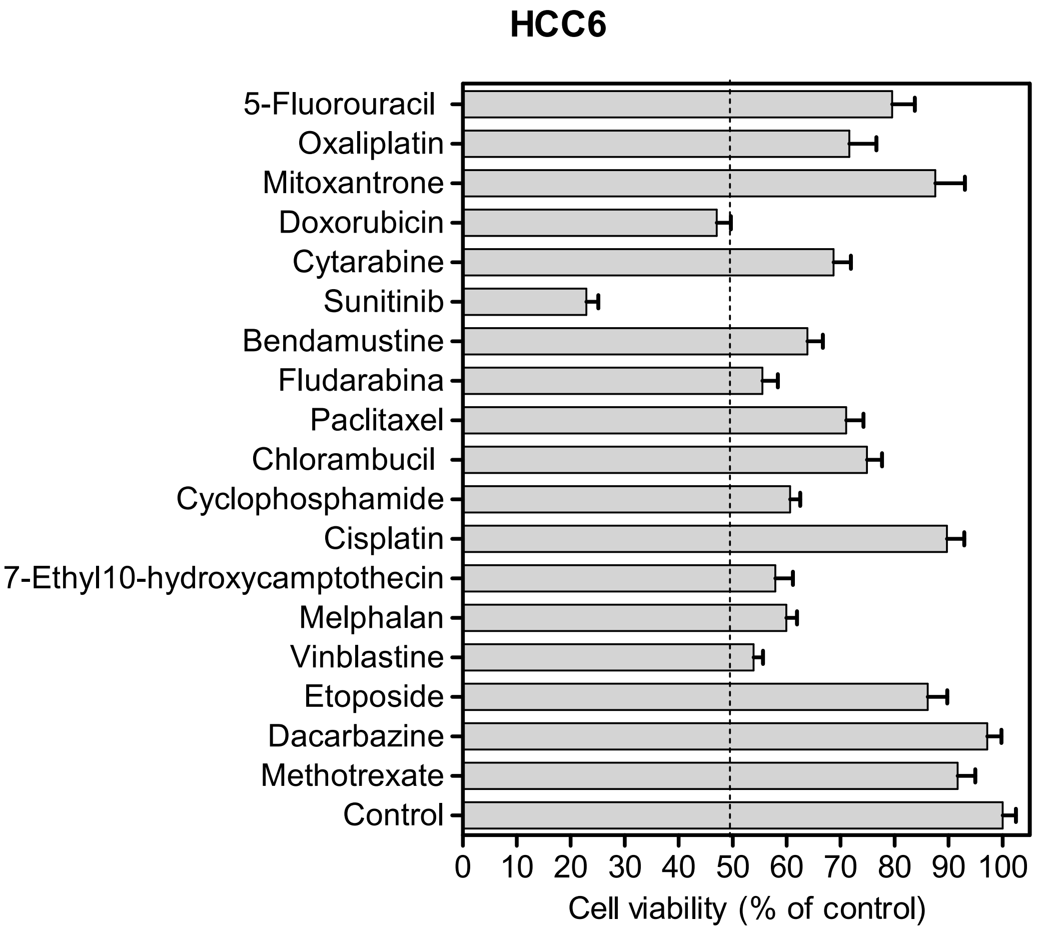

Supplementary Figure 8. Chemosensitivity of the HCC6 liver cancer patient. Cell viability was determined using the alamar blue method after 72 h of incubation with a panel of 18 drugs each at a concentration of 25 μg/mL. An inhibition rate greater than 50% was defined as the sensitivity value for evaluating the drug. The data are shown as the mean ± S.E.M. of eight replicates.

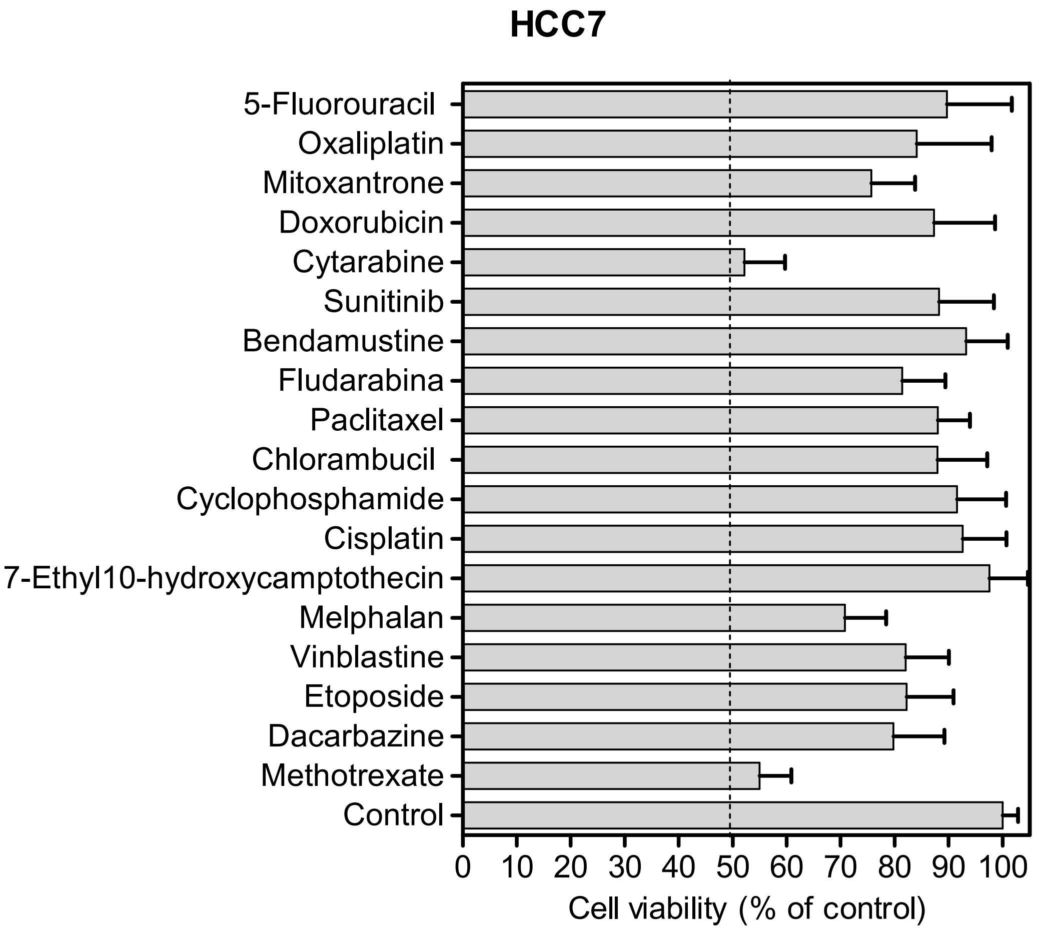

Supplementary Figure 9. Chemosensitivity of the HCC7 liver cancer patient. Cell viability was determined using the alamar blue method after 72 h of incubation with a panel of 18 drugs each at a concentration of 25 μg/mL. An inhibition rate greater than 50% was defined as the sensitivity value for evaluating the drug. The data are shown as the mean ± S.E.M. of eight replicates.

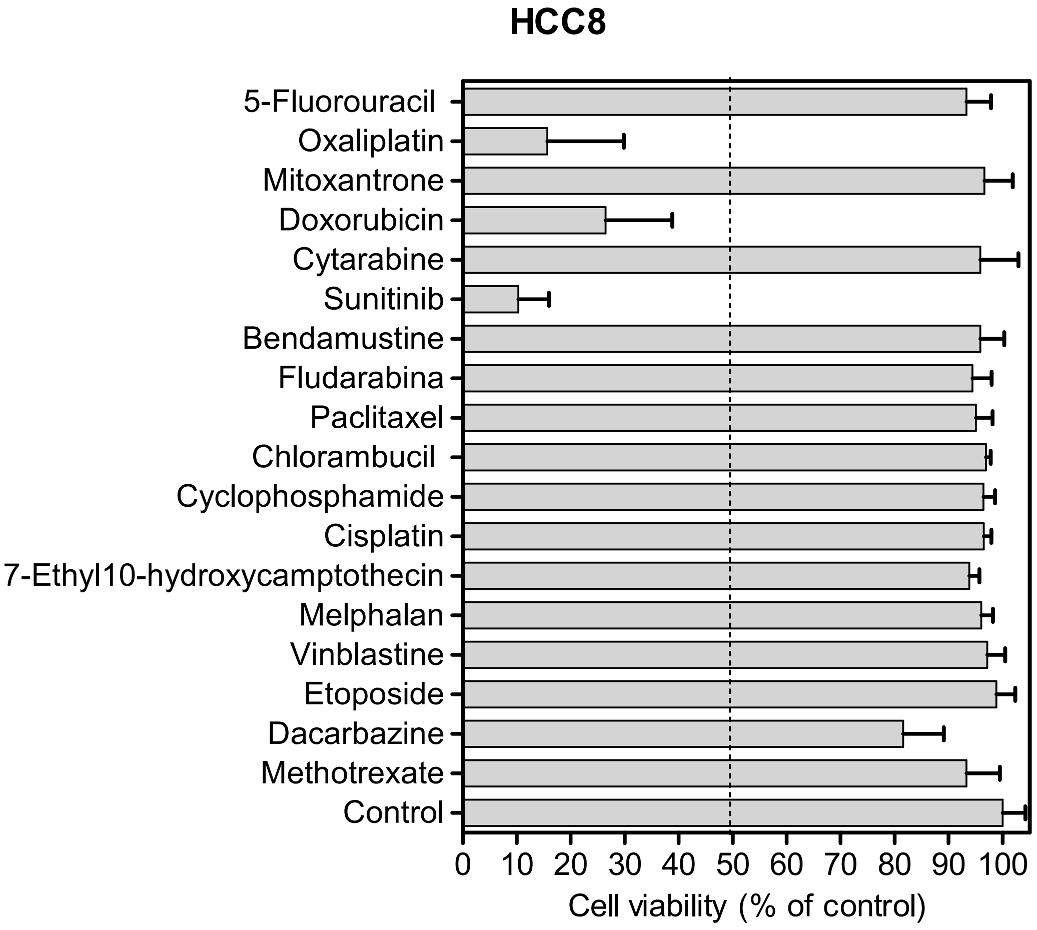

Supplementary Figure 10. Chemosensitivity of the HCC8 liver cancer patient. Cell viability was determined using the alamar blue method after 72 h of incubation with a panel of 18 drugs each at a concentration of 25 μg/mL. An inhibition rate greater than 50% was defined as the sensitivity value for evaluating the drug. The data are shown as the mean ± S.E.M. of eight replicates.

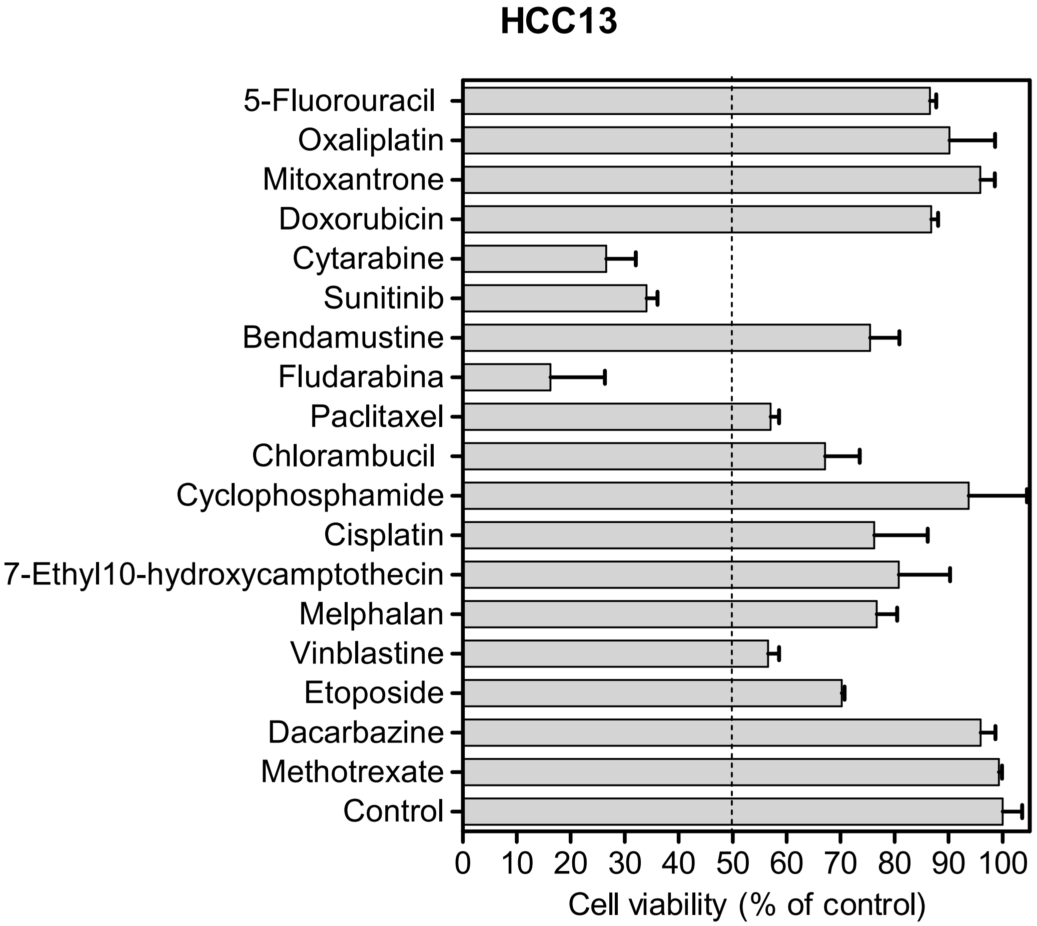

Supplementary Figure 11. Chemosensitivity of the HCC13 liver cancer patient. Cell viability was determined using the alamar blue method after 72 h of incubation with a panel of 18 drugs each at a concentration of 25 µg/mL. An inhibition rate greater than 50% was defined as the sensitivity value for evaluating the drug. The data are shown as the mean ± S.E.M. of eight replicates.

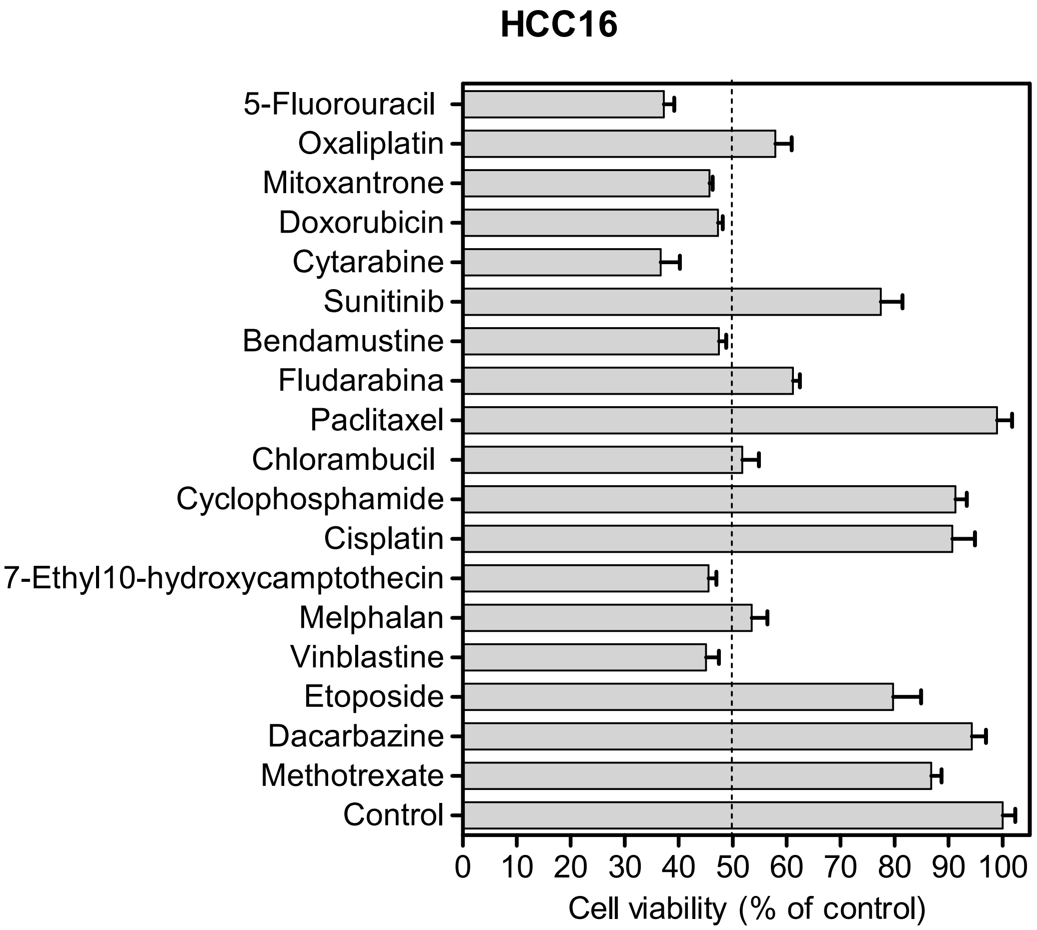

Supplementary Figure 12. Chemosensitivity of the HCC16 liver cancer patient. Cell viability was determined using the alamar blue method after 72 h of incubation with a panel of 18 drugs each at a concentration of 25 μg/mL. An inhibition rate greater than 50% was defined as the sensitivity value for evaluating the drug. The data are shown as the mean ± S.E.M. of eight replicates.

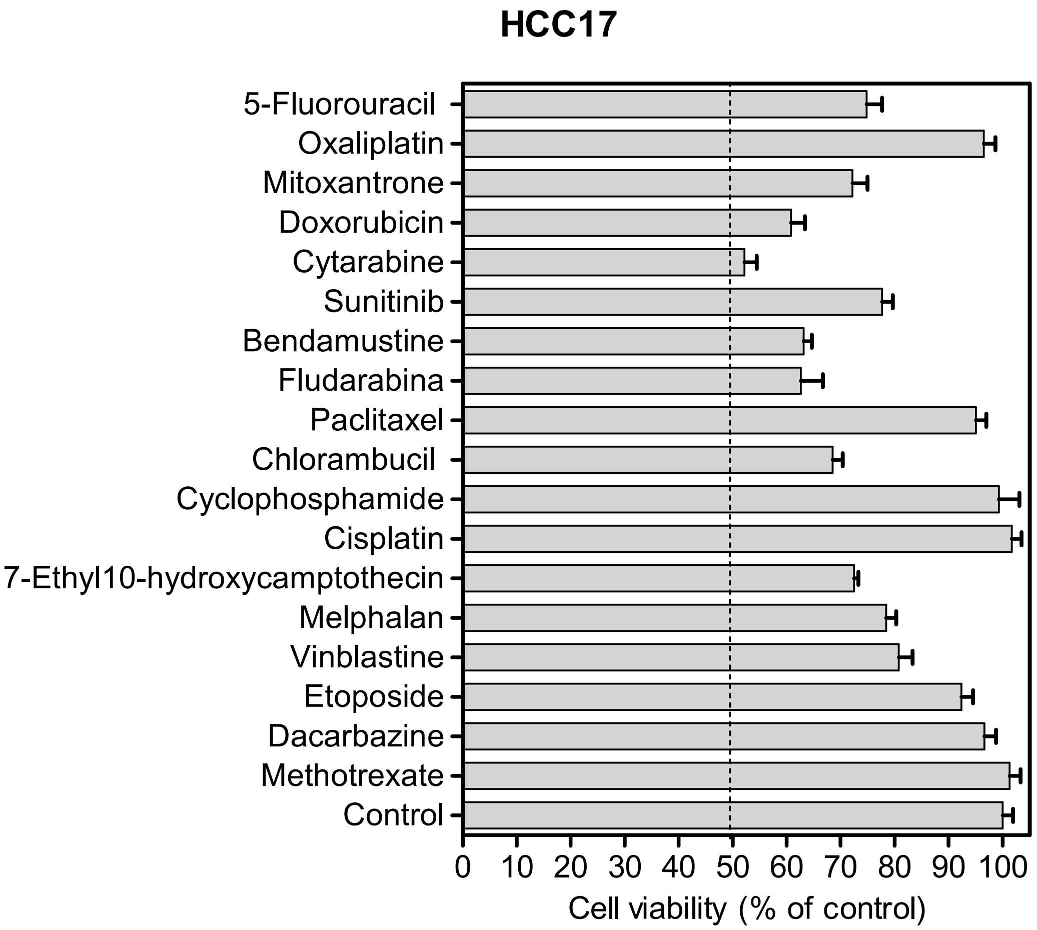

Supplementary Figure 13. Chemosensitivity of the HCC17 liver cancer patient. Cell viability was determined using the alamar blue method after 72 h of incubation with a panel of 18 drugs each at a concentration of 25 µg/mL. An inhibition rate greater than 50% was defined as the sensitivity value for evaluating the drug. The data are shown as the mean ± S.E.M. of eight replicates.

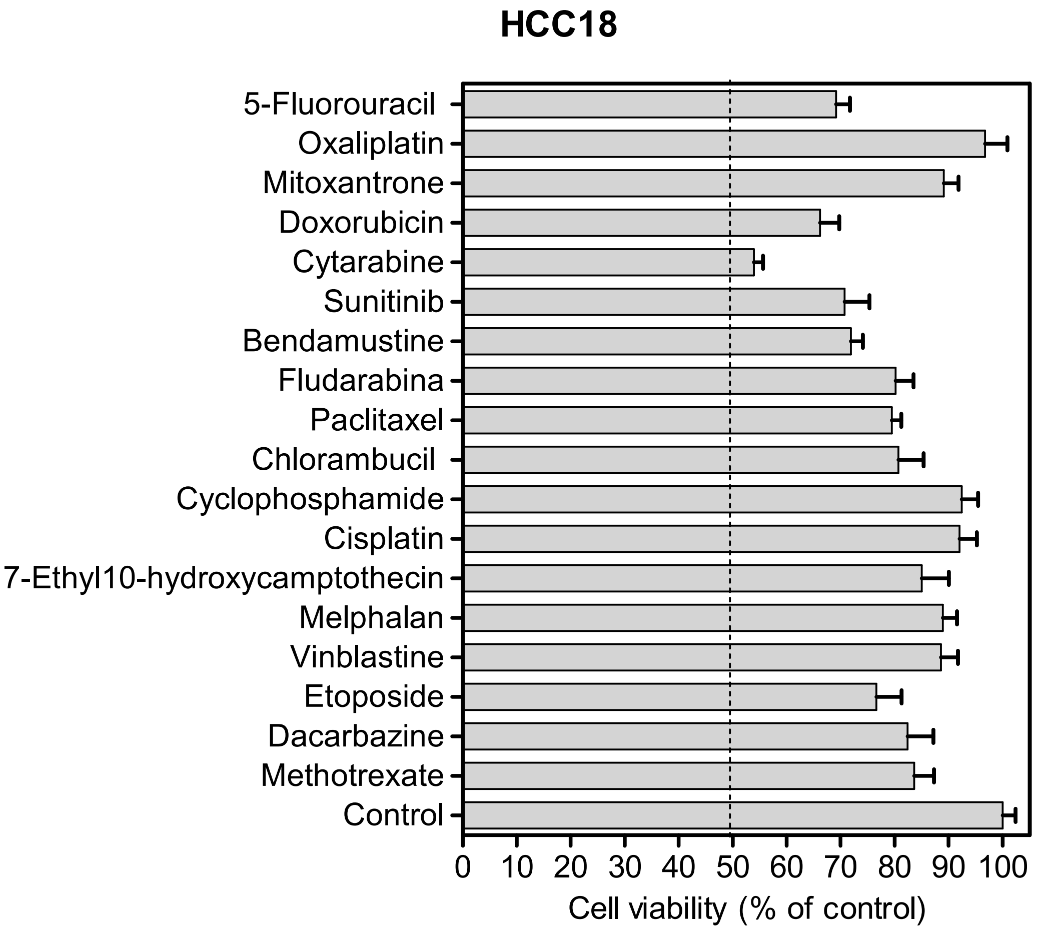

Supplementary Figure 14. Chemosensitivity of the HCC18 liver cancer patient. Cell viability was determined using the alamar blue method after 72 h of incubation with a panel of 18 drugs each at a concentration of 25 μg/mL. An inhibition rate greater than 50% was defined as the sensitivity value for evaluating the drug. The data are shown as the mean ± S.E.M. of eight replicates.

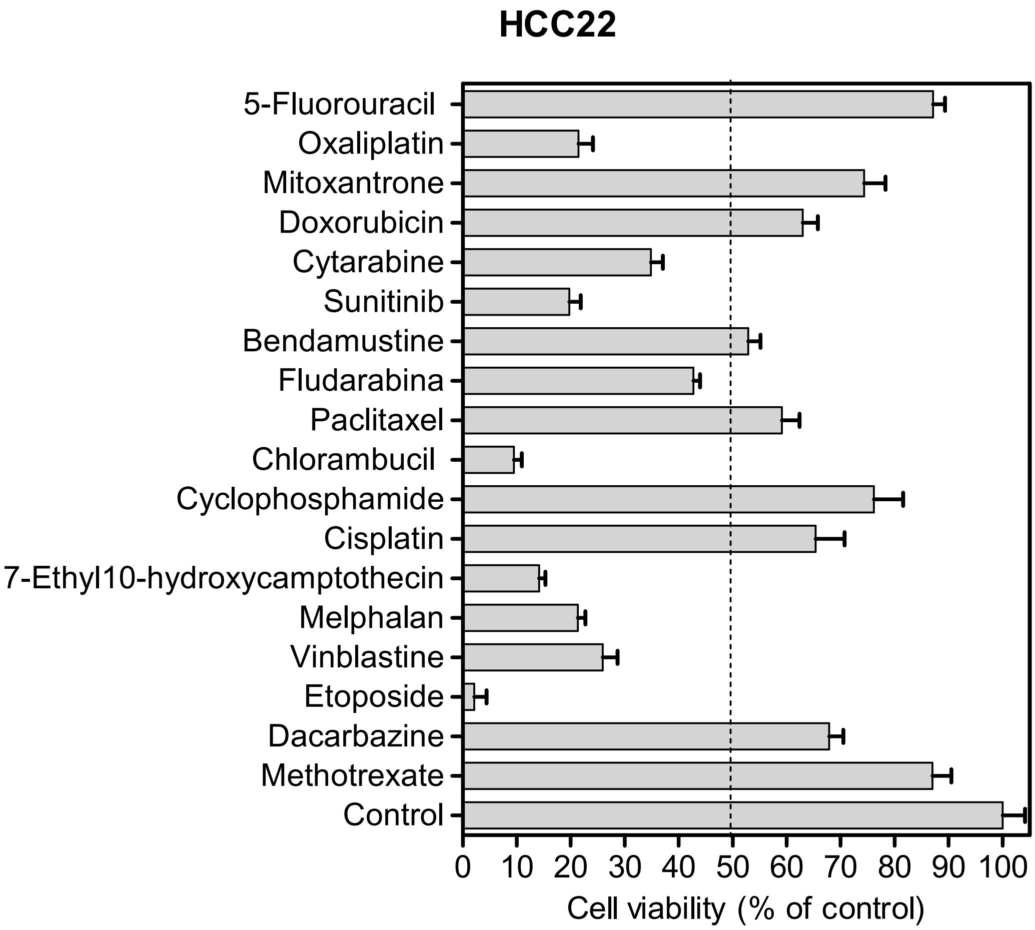

Supplementary Figure 15. Chemosensitivity of the HCC22 liver cancer patient. Cell viability was determined using the alamar blue method after 72 h of incubation with a panel of 18 drugs each at a concentration of 25 μg/mL. An inhibition rate greater than 50% was defined as the sensitivity value for evaluating the drug. The data are shown as the mean ± S.E.M. of eight replicates.

Supplementary Table 1. Fold change of the expression of *PTCH1*, *GLI1*, *GLI2* and *GLI3* genes in patients with liver cancer

**Cases**	**Gene (Fold change)**											
	PTCH1			***GLI1***			***GLI2***			***GLI3***		
	HCC	TM	NNL	HCC	TM	NNL	HCC	TM	NNL	HCC	TM	NNL
**HCC5**	1.72	0.44	0.00	21.68	0.00	0.00	33.34	4.11	0.00	3.91	0.45	0.00
**HCC6**	0.34	0.34	0.27	0.00	0.74	0.00	0.00	0.00	0.00	1.87	0.33	0.21
**HCC7**	0.36	0.24	0.64	0.00	0.47	0.53	0.00	0.75	1.45	0.00	0.47	0.45
**HCC8**	0.21	0.00	0.54	0.76	0.00	0.25	0.49	0.00	2.53	0.46	0.00	0.89
**HCC13**	1.09	0.96	1.03	1.20	1.39	0.74	5.83	1.40	1.03	3.24	0.77	0.53
**HCC16**	0.33	0.28	4.93	0.00	1.04	0.87	0.00	2.08	1.34	0.30	0.33	0.48
**HCC17**	0.85	0.47	1.22	0.12	2.65	2.24	1.29	0.95	2.25	0.00	0.30	0.91
**HCC18**	0.93	0.00	1.64	2.37	0.00	3.17	21.37	0.00	13.38	6.95	0.00	3.30
**HCC22**	0.52	0.00	1.18	1.50	0.00	0.00	10.09	0.00	0.00	3.00	0.00	0.00

TM, tumor lateral margin at the interface with the nonneoplastic liver.

NNL, distant nonneoplastic liver tissue far from the tumor.

Supplementary Table 2. Antibodies used

Epitope	Clone	Dilution	Catalog number	Manufacturer
Hep-Par	OCH1E5	Ready to use	GA62461-2	DAKO (Agilent)
Arginase	Polyclonal	1:500	ABS535	Sigma
Glutamine syntase	GS-6	Ready to use	7107757001	Roche
pCEA	II-7	Ready to use	GA62261-2	DAKO (Agilent)
CK7	OV-TL 12/30	Ready to use	IR61961-2	DAKO (Agilent)
CK19	RCK108	Ready to use	IR61561-2	DAKO (Agilent)
EpCam	Ber-EP4	Ready to use	GA63761-2	DAKO (Agilent)
CD56	123C3	Ready to use	IR62861-2	DAKO (Agilent)
GLI1	Polyclonal	Ready to use	NB600-600	Novus Biologicals
